# Transcriptome analysis explores genes related to shikonin biosynthesis in Lithospermeae plants and provides insights into Boraginales’ evolutionary history

**DOI:** 10.1038/s41598-017-04750-1

**Published:** 2017-06-30

**Authors:** Feng-Yao Wu, Cheng-Yi Tang, Yu-Min Guo, Zhuo-Wu Bian, Jiang-Yan Fu, Gui-Hua Lu, Jin-Liang Qi, Yan-Jun Pang, Yong-Hua Yang

**Affiliations:** State Key Laboratory of Pharmaceutical Biotechnology, NJU-NJFU Joint Institute of Plant Molecular Biology, School of Life Sciences, Nanjing University, Nanjing, 210093 China

## Abstract

Shikonin and its derivatives extracted from Lithospermeae plants’ red roots have current applications in food and pharmaceutical industries. Previous studies have cloned some genes related to shikonin biosynthesis. However, most genes related to shikonin biosynthesis remain unclear, because the lack of the genome/transcriptome of the Lithospermeae plants. Therefore, in order to provide a new understanding of shikonin biosynthesis, we obtained transcriptome data and unigenes expression profiles in three shikonin-producing Lithospermeae plants, i.e., *Lithospermum erythrorhizon*, *Arnebia euchroma* and *Echium plantagineum*. As a result, two unigenes (i.e., *G10H* and *12OPR*) that are involved in “shikonin downstream biosynthesis” and “methyl jasmonate biosynthesis” were deemed to relate to shikonin biosynthesis in this study. Furthermore, we conducted a Lamiids phylogenetic model and identified orthologous unigenes under positive selection in above three Lithospermeae plants. The results indicated Boraginales was more relative to Solanales/Gentianales than to Lamiales.

## Introduction

Shikonin and its derivatives, red naphthoquinones, are widely found in the epidermis of the roots of the Lithospermeae plants, such as genera *Lithospermum*, *Arnebia* and *Echium* ^1–3^. In particular, *Lithospermum erythrorhizon*, *Arnebia euchroma* and *Echium plantagineum*, which are known as “alkanets” or “gromwells”, were originally used as natural dyes and herbal medicines in both Europe and the Orient for many centuries^1–3^. Shikonin and its derivatives are currently used in the food industry, cosmetics production and modern pharmaceutical synthesis based on their various biological activities, such as antioxidant and antibacteria^1–3^. Recently, it was reported that shikonin and its derivatives could induce apoptosis of many type cancer cells and perform anticancer activities^4, 5^. To meet the increasing demand, numerous attempts to synthesize shikonin and its derivatives have been invented and reported, including attempts using chemosynthesis and two-stage culture biosynthesis^1–3^.

According to previous studies, the proposed biosynthesis of the shikonin and its derivatives stems from mevalonate biosynthesis (terpenoid backbone biosynthesis) and phenylpropanoid biosynthesis^1, 6, 7^. In the past 30 years, several genes that encoding enzymes directly involved in the shikonin biosynthesis, such as hydroxymethylglutaryl-CoA synthase gene (*HMGS*), hydroxymethylglutaryl-CoA reductase gene (*HMGR*), phenylalanine ammonia lyase (*PAL*), cinnamate 4-monooxygenase (*C4H*) and p-hydroxybenzoate geranyltransferase gene (*PGT*), have been cloned and characterized in *L*. *erythrorhizon* or *A*. *euchroma* ^8–12^. In addition, several genes indirectly related to shikonin biosynthesis have been discovered and cloned, such as *L*. *erythrorhizon* dark-inducible gene *1*, *2*, *3*, *4*, *5a/b/c* (*LeDI*-*1*, *2*, *3*, *4*, *5a/b/c*) and *L*. *erythrorhizon* 1-aminocyclopropane-1-carboxylate synthase gene (*LeACS*)^12–14^. Moreover, several factors, such as light, methyl jasmonate, nitric oxide, ethylene and fungal elicitor are crucial regulators of shikonin biosynthesis^12, 15–19^. However, the complete pathway of the shikonin biosynthesis remains obscure, and most genes directly/indirectly related to shikonin biosynthesis remain unclear, particularly the enzymes/genes that participate in the shikonin downstream biosynthesis (i.e., the pathway from geranyl-hydroxy-benzoate to shikonin) and the biosynthesis of the regulatory factors (e.g., methyl jasmonate, nitric oxide, ethylene)^1, 2^.

At present, RNA sequencing (RNA-Seq) is an effective platform to acquire transcriptome information and gene sequences at a minimal cost compared with whole genome *de novo* sequencing. This technology provides new opportunities to explore and identify novel genes involved in natural product biosynthesis in plants. For instance, Zhang *et al*. constructed gene co-expression networks related to *D*-myo-inositol phosphates (IP) in maize by integrating transcriptome and metabolite data, and discovered three new genes related to IP metabolism^20^. Dugé *et al*. constructed an optimized transcriptomic resource for *Catharanthus roseus* by processing previous published transcriptome data, and provided new perspectives for the understanding of the monoterpenoid indole alkaloids biosynthesis (MIA) in *C*. *roseus* ^21^. In addition, RNA-Seq also provides new opportunities to perform multiple comparisons and phylogenetic analysis to discover genic molecular evolution in different species. For example, Yang *et al*. found that similar functional categories had undergone positive selection in high-altitude *Phrynocephalus* and *Rana* species by comparing transcriptome data from different low-/high-altitude poikilothermic species^22^. Wickett *et al*. assessed phylogenetic models to explore the origin and early diversification of land plants by using 92 plant transcriptome data plus 11 public plant genome data^23^.

Therefore, in order to provide a new understanding of shikonin biosynthesis, we performed transcriptome analysis for three Lithospermeae plants, i.e., *L*. *erythrorhizon*, *A*. *euchroma* and *E*. *plantagineum*. First, we detected shikonin and its derivatives in their green leaves/stems (GL, non-producing shikonin) and red roots (RR, producing shikonin) by high performance liquid chromatography (HPLC). Subsequently, we constructed their transcriptome libraries and compared unigenes expression profiles between GL and RR lines in the above three Lithospermeae plants. The results indicated that two unigenes (i.e., *G10H* and *12OPR*) were related to shikonin biosynthesis in this present study. Furthermore, we conducted a Lamiids phylogenetic model based on above three Lithospermeae plants’ transcriptome data plus seven public plant genome data. The results showed that Boraginales might be resolved as a sister to Solanales/Gentianales rather than Lamiales. Moreover, we identified orthologous unigenes under positive selection (PSOs) in the above three Lithospermeae plants, and found that one PSO (i.e., *4CL*) potentially influenced shikonin biosynthesis in this study.

## Results and Discussion

### Detection of shikonin and its derivatives in GL and RR lines by HPLC

**Figure 1:**
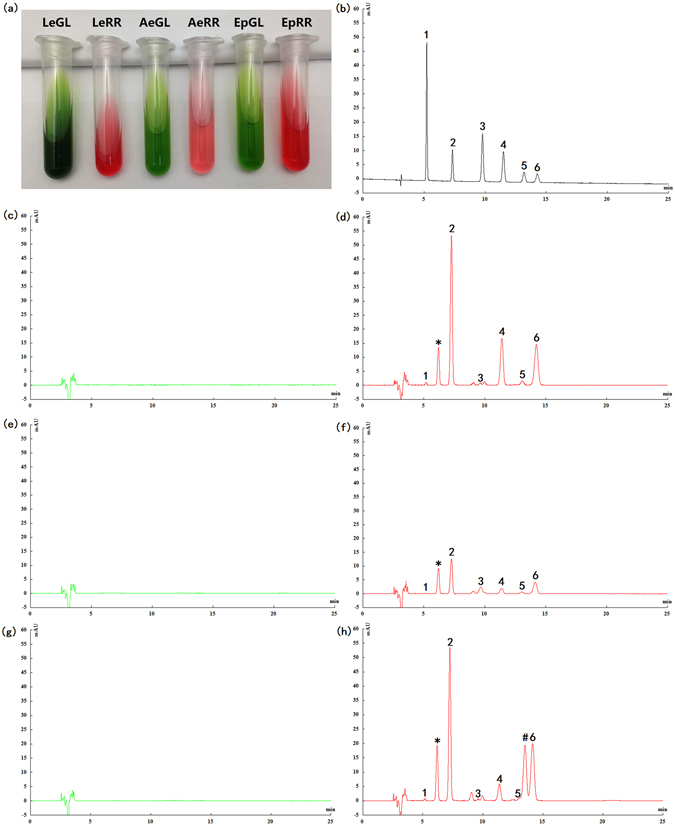
Detection of shikonin and its derivatives in the GL and RR lines of the three Lithospermeae plants (i.e., *Lithospermum erythrorhizon*, *Arnebia euchroma* and *Echium plantagineum*) by HPLC. (**a**) The ethanol extracts from the above three Lithospermeae plants, (**b**) standards, (**c**) LeGL, (**d**) LeRR, (**e**) AeGL, (**f**) AeRR, (**g**) EpGL, (**h**) EpRR; 1. Shikonin, 2. Acetylshikonin, 3. Deoxyshikonin, 4. Isobutylshikonin; 5. *β*, *β*’-Dimethylacrylshikonin, 6. *α*-Methyl-n-butyrylshikonin/Isovalerylshikonin (isomerized products can not be separated in this study). ^*^ *β*-Hydroxyisovalerylshikonin (inferential). ^#^Unknown compound.

Detection of shikonin and its derivatives in GL and RR lines from three Lithospermeae plants, i.e., *L*. *erythrorhizon*, *A*. *euchroma* and *E*. *plantagineum*, was performed using HPLC. The results shown that shikonin and its derivatives scarcely existed in all GL lines (Fig. 1a,b,c,e and g); expectedly, shikonin and its derivatives, e.g., shikonin, acetylshikonin, and isobutyrylshikonin, were detected in all RR lines as previously reported (Fig. 1a,b,d,f and h; Supplementary Table S1)^24–26^. Furthermore, we deduced that the peak* might be *β*-hydroxyisovalerylshikonin, which is consistent with the previous studies^24–26^. In summary, shikonin biosynthesis should exist only in the RR lines rather than GL lines in the three Lithospermeae plants assessed in this present study (Fig. 1 and Supplementary Table S1).

### Transcriptome sequencing, assembly, annotation and KEGG classification

**Figure 2:**
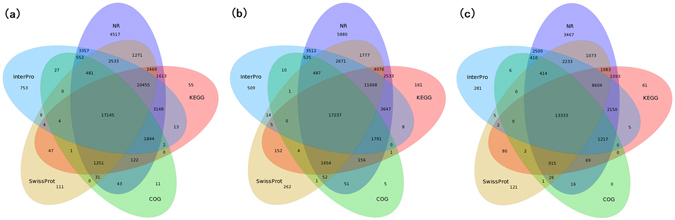
The Venn diagram of unigenes annotation from five public protein databases (The colors severally denote NR, SwissProt, KEGG, InterPro, COG databases). (**a**) *Lithospermum erythrorhizon*, (**b**) *Arnebia euchroma*, (**c**) *Echium plantagineum*.

To obtain an overview of genes associated with shikonin biosynthesis, six cDNA libraries (i.e., LeGL, LeRR, AeGL, AeRR, EpGL and EpRR) were prepared from the GL and RR lines of the three Lithospermeae plants, and then were sequenced on an Illumina HiSeq™ 4000 platform. After raw data filtration and *de novo* assembly, 76455 unigenes with total length of 84.51 (Mb) and N50 length (median length of all non-redundant sequences) of 1856 (bp) were generated in the *L*. *erythrorhizon* libraries; and 89639 unigenes with total length of 87.19 (Mb) and N50 length of 1629 (bp) were generated in the *A*. *euchroma* libraries; and 54627 unigenes with total length of 55.59 (Mb) and N50 length of 1653 (bp) were generated in the *E*. *plantagineum* libraries (Table 1). Subsequently, the functional annotation of all assembled unigenes was performed using seven public databases, i.e., Non-redundant Protein Sequence (NR), Nucleotide Sequence (NT), SwissProt, InterPro, Kyoto Encyclopedia of Genes and Genomes (KEGG), Clusters of Orthologous Groups of Proteins (COG) and Gene Ontology (GO). As a result, a total of 54128 unigenes (70.80%) in the *L*. *erythrorhizon* libraries, 61221 unigenes (68.30%) in the *A*. *euchroma* libraries and 41313 unigenes (75.62%) in the *E*. *plantagineum* libraries were annotated in at least one of the above databases (Table 1). Furthermore, we synthesized annotation information from five protein databases (i.e., NR, SwissProt, KEGG, InterPro, COG). The results indicated that a total of 17145 unigenes (22.42%) in the *L*. *erythrorhizon* libraries, 17237 unigenes (19.23%) in the *A*. *euchroma* libraries and 13333 unigenes (24.41%) in the *E*. *plantagineum* libraries were conjointly annotated by all five protein databases (Fig. 2). In addition, all assembled unigenes were performed coding sequence (CDS) prediction. As a result, a total of 53,675 (70.20%), 60728 (67.75%), 40441 (74.03%) CDS were generated in the above three Lithospermeae plants, respectively (Table 1). Subsequently, we classified all unigenes based on their function in KEGG database to better explore and understand unigenes potentially related to shikonin biosynthesis because KEGG is a highly integrated protein database designed to link genes to gene products (mostly proteins) in the metabolic pathways. As a result, a total of 38173 unigenes (49.93%) in the *L*. *erythrorhizon*, 43093 unigenes (48.07%) in the *A*. *euchroma* and 29514 unigenes (54.03%) in the *E*. *plantagineum* were assigned to 126 pathways, which were grouped into 20 sub categories and 7 categories (Supplementary Table S2).

**Table 1:** Overview of transcriptome assembly, annotation and CDS prediction.

		*Lithospermum erythrorhizon*	*Arnebia euchroma*	*Echium plantagineum*
Assembly	Total Length (bp)	84,508,068	87,189,611	55,592,752
	N50 Length (bp)	1,856	1,629	1,653
	GC (%)	39.84	40.62	40.76
	Total Unigenes	76,455	89,639	54,627
Annotation	NR	50,832	57,717	39,503
	NT	41,021	45,267	32,705
	SwissProt	35,812	40,061	28,792
	InterPro	40,327	42,085	31,160
	KEGG	38,173	43,093	29,514
	COG	21,514	21,975	16,409
	GO	7,486	10,182	7,871
	Overall	54,128	61,221	41,313
CDS prediction	Blast CDS	50,396	56,602	38,863
	ESTScan CDS	3,279	4,126	1,578
	Total CDS	53,675	60,728	40,441

### Differentially expressed unigene (DEG) identification and KEGG enrichment

**Figure 3:**
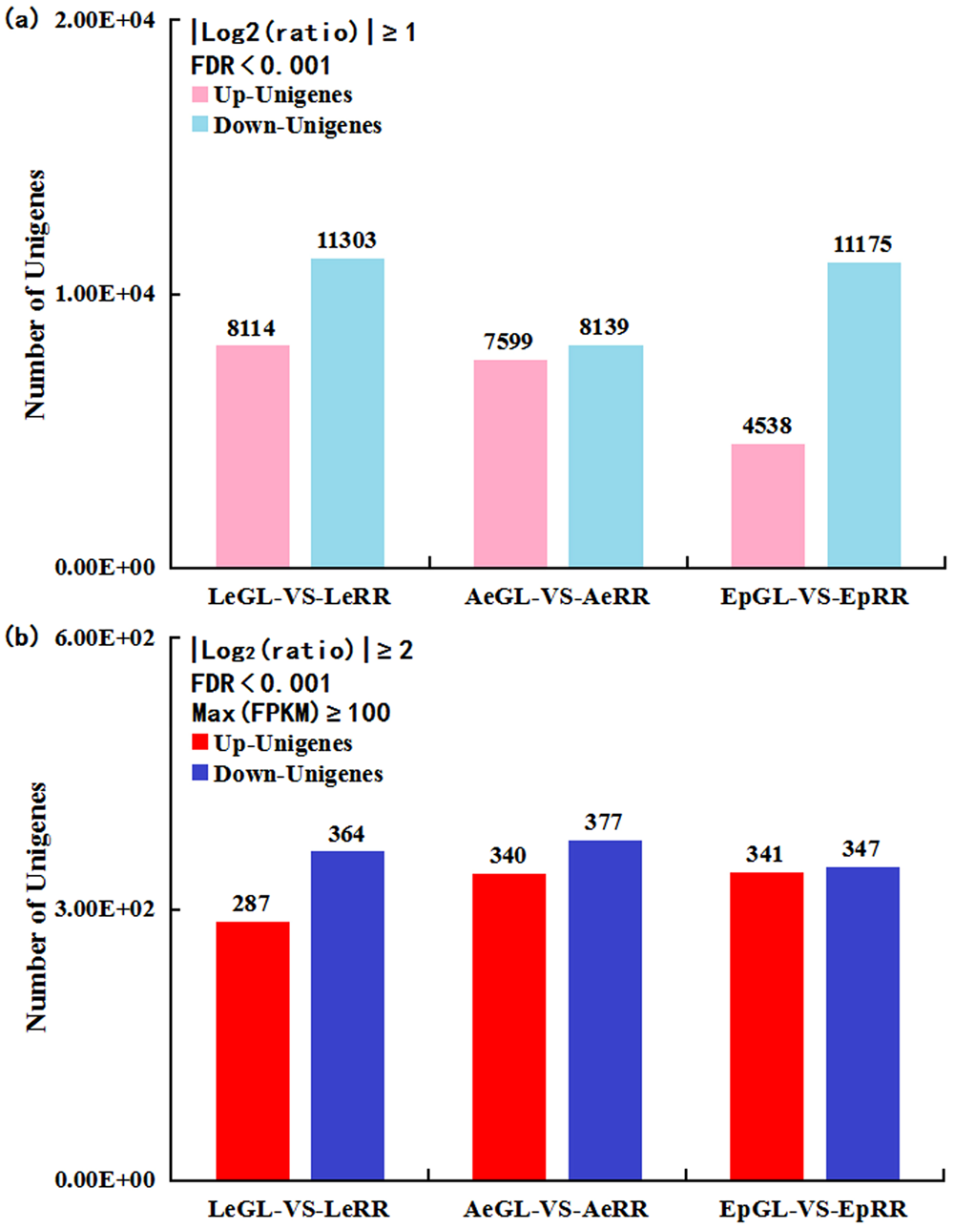
Unigene expression between GL and RR lines in *Lithospermum erythrorhizon*, *Arnebia euchroma* and *Echium plantagineum*. (**a**) Significantly differentially expressed unigenes (DEGs); (**b**) Highly significant differentially expressed unigenes (HDEGs).

According to the general thresholds (i.e., |Log2(ratio)| ≥ 1 and FDR < 0.001), a total of 19447 unigenes were significantly differentially expressed in the *L*. *erythrorhizon* libraries, including 8144 up-expressed and 11303 down-expressed in the LeRR line; and a total of 15738 unigenes were significantly differentially expressed in the *A*. *euchroma* libraries, including 7599 up-expressed and 8139 down-expressed in the AeRR line, and a total of 15713 unigenes were significantly differentially expressed in the *E*. *plantagineum* libraries including 4538 up-expressed and 11175 down-expressed in the EpRR line (Fig. 3a). Subsequently, we classified all DEGs into KEGG classification using the same method and performed KEGG enrichment analysis for the pathways associated with each DEG. As a result, a total of 44 pathways in the *L*. *erythrorhizon* libraries, 45 pathways in the *A*. *euchroma* libraries and 22 pathways in the *E*. *plantagineum* libraries were significant enriched (Supplementary Table S2). Specifically, these enriched pathways mainly referred to “energy metabolism”, “carbohydrate metabolism”, “biosynthesis of other secondary metabolites” and “metabolism of terpenoids and polyketides” pathway sub-categories, particularly several pathways involved in “starch and sucrose metabolism”, “photosynthesis”, “phenylpropanoid biosynthesis” and “terpenoid backbone biosynthesis” (Supplementary Table S2). Therefore, KEGG enrichment revealed that photosynthesis metabolism and shikonin biosynthesis were the main distinctions between the RR and GL lines of the above three Lithospermeae plants. However, the DEGs generated according to the general thresholds were seemingly too numerous; thus, it was difficult to identify the major genes related to shikonin biosynthesis. Therefore, we defined highly significant differentially expressed unigenes (HDEGs) on the basis of the stringent thresholds (i.e., |Log2(ratio)| ≥ 1 and FDR < 0.001 and Max(FPKM) ≥ 100) in this present study. As a result, a total of 651 HDEGs including 287 up-expressed and 364 down-expressed were generated in the LeRR line of the *L*. *erythrorhizon* libraries; and a total of 717 HDEGs including 340 up-expressed and 377 down-expressed were generated in the AeRR line of the *A*. *euchroma* libraries; and a total of 688 HDEGs including 341 up-expressed and 347 down-expressed were generated in the EpRR line of the *E*. *plantagineum* libraries (Supplementary Table S3, Fig. 3b).

### Novel unigenes related to shikonin biosynthesis

According to the stringent thresholds, we found that a series of unigenes that were previously reported to be related to shikonin biosynthesis are up-expressed in all RR lines, such as *HMGR*, *PAL*, *PGT*, *LeDI*-*1*, *LePR2* genes and so on (Supplementary Table S3). Moreover, we also identified two novel unigenes (i.e. *G10H* and *12OPR*) related to shikonin biosynthesis in this present study (Supplementary Table S3).

**Figure 4:**
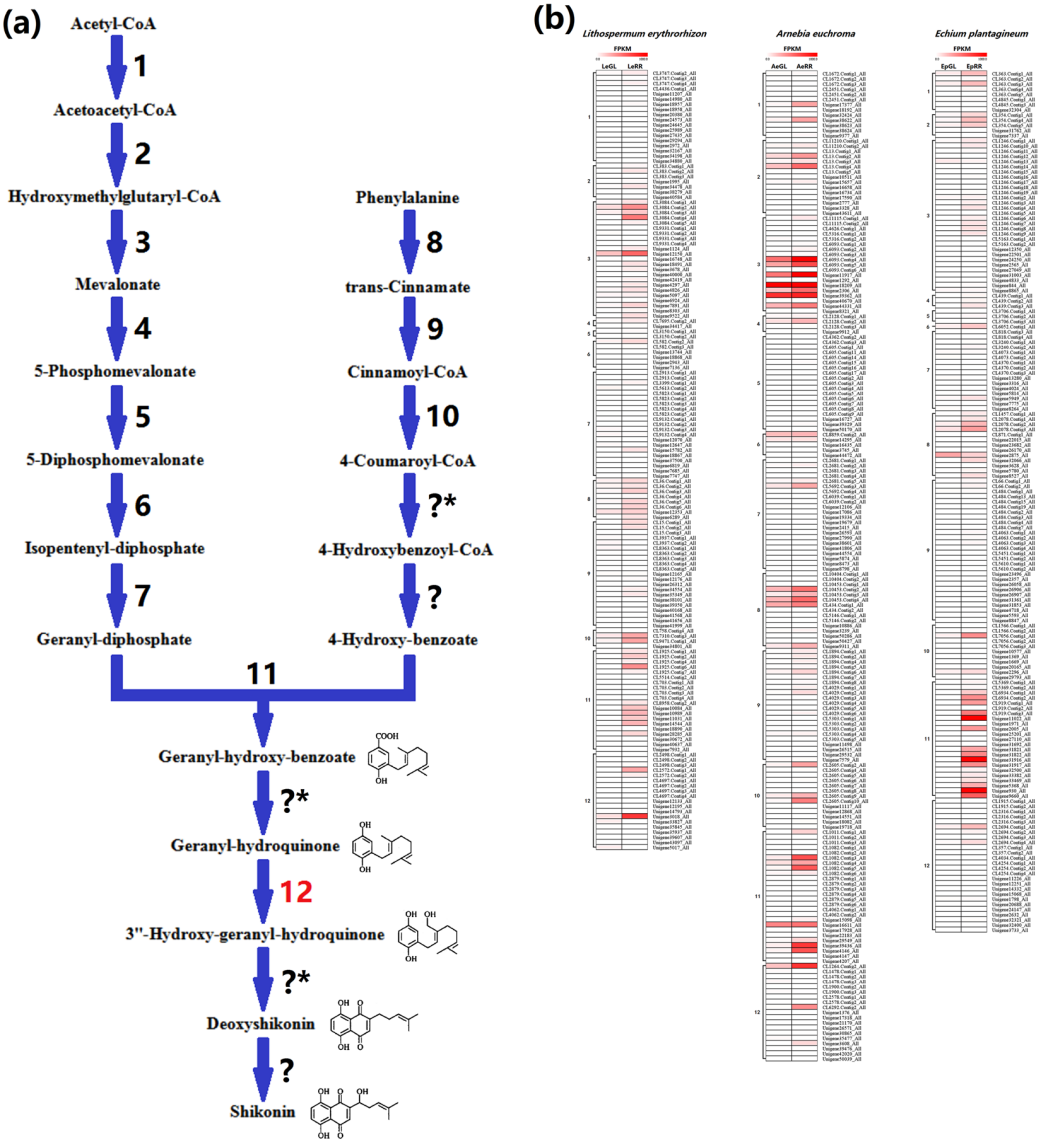
Unigene expression involved in shikonin biosynthesis in *Lithospermum erythrorhizon*, *Arnebia euchroma* and *Echium plantagineum*. (**a**) Shikonin biosynthesis; (**b**) Unigene expressions involved in shikonin biosynthesis? One step unknown?* Several steps unknown; 1. Acetoacetyl-coenzyme A thiolase gene (*AACT*), 2. Hydroxy-methylglutaryl CoA synthase gene (*HMGS*), 3. Hydroxy-methylglutaryl-CoA reductase gene (*HMGR*), 4. Mevalonate kinase gene (*MK*), 5. Phosphomevalonate kinase gene (*PMK*), 6. Mevalonate disphosphate decarboxylase gene (*MVD*), 7. Geranyl diphosphate synthase gene (*GDS*), 8. Phenylalanine ammonia-lyase gene (*PAL*), 9. 4-Coumarate:CoA ligase gene (*4CL*), 10. Cinnamic acid 4-hydroxylase gene (*C4H*), 11. p-Hydroxybenzoate geranyltransferase gene (*PGT*), 12. Geraniol 10-hydroxylase gene (*G10H*, predicted).

First, we found that a geraniol 10-hydroxylase unigene (*G10H*; P-450 monooxygenase) is up-regulated in all RR lines (Supplementary Tables S3 and S4, Fig. 4). In addition, Yamamoto *et al*. reported a geranyl-hydroquinone 3″-hydroxylase enzyme (G3″H) that participates in shikonin biosynthesis as a P-450 monooxygenase, but they failed to report its nucleotide and amino acid sequence^27^. Therefore, we deduced that the *G10H* in this present study might be the gene of G3″H, which participates in shikonin downstream biosynthesis, because the G10H and G3″H enzyme have a similar function and belong to the P-450 monooxygenase family^27, 28^. The reason why that *G3*″*H* was annotated as *G10H* in this present study might be the lack of the gene or protein information of the *G3*″*H/*G3″H in the above public databases.

**Figure 5:**
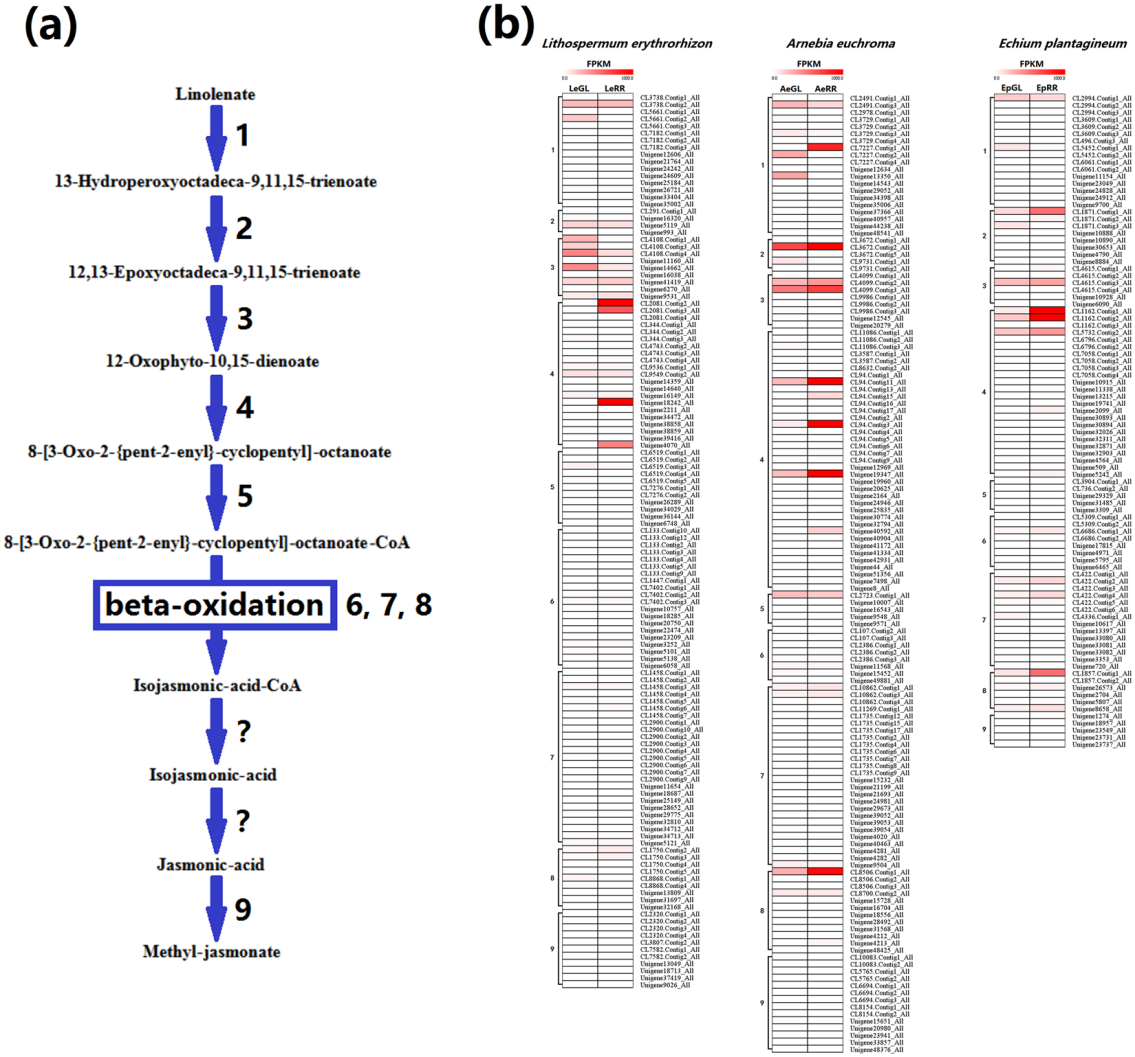
Unigene expression involved in methyl jasmonate biosynthesis in *Lithospermum erythrorhizon*, *Arnebia euchroma* and *Echium plantagineum*. (**a**) Methyl jasmonate biosynthesis, (**b**) Unigene expression involved in methyl jasmonate biosynthesis? One step unknown; 1. Lipoxygenase gene (*LOX*), 2. Allene oxide synthase gene (*AOS*), 3. Allene oxide cyclase gene (*AOC*), 4. 12-Oxophytodienoate reductase gene (*12OPR*), 5. CoA ligase gene (*CL*), 6. Acyl-CoA oxidase gene (*ACX*), 7. Fatty acid β-oxidation multifunctional protein MFP/AIM gene (*MFP/AIM*), 8. 3-ketoacyl-CoA thiolase gene (*KAT*), 9. Jasmonate O-methyltransferase gene (*JMT*).

Second, we found that *LeDI*-*5a* and *LeDI*-*5b*, which were specifically highly expressed in all RR lines as previously reported^12^, were re-annotated as the 12-oxophytodienoate reductase gene (*12OPR*) (Supplementary Tables S3 and S5, Fig. 5). The 12OPR protein is a key enzyme in methyl jasmonate biosynthesis (Supplementary Table S5, Fig. 5)^29, 30^. In addition, previous studies reported that exogenous methyl jasmonate could improve shikonin biosynthesis^15, 16^. Thus, we speculated that up-expressed *12OPR* unigenes (i.e., *LeDI*-*5a/b* gene) could promote MeJA biosynthesis in the above Lithospermeae plants, and endogenous MeJA accumulation could further promote shikonin biosynthesis.

**Figure 6:**
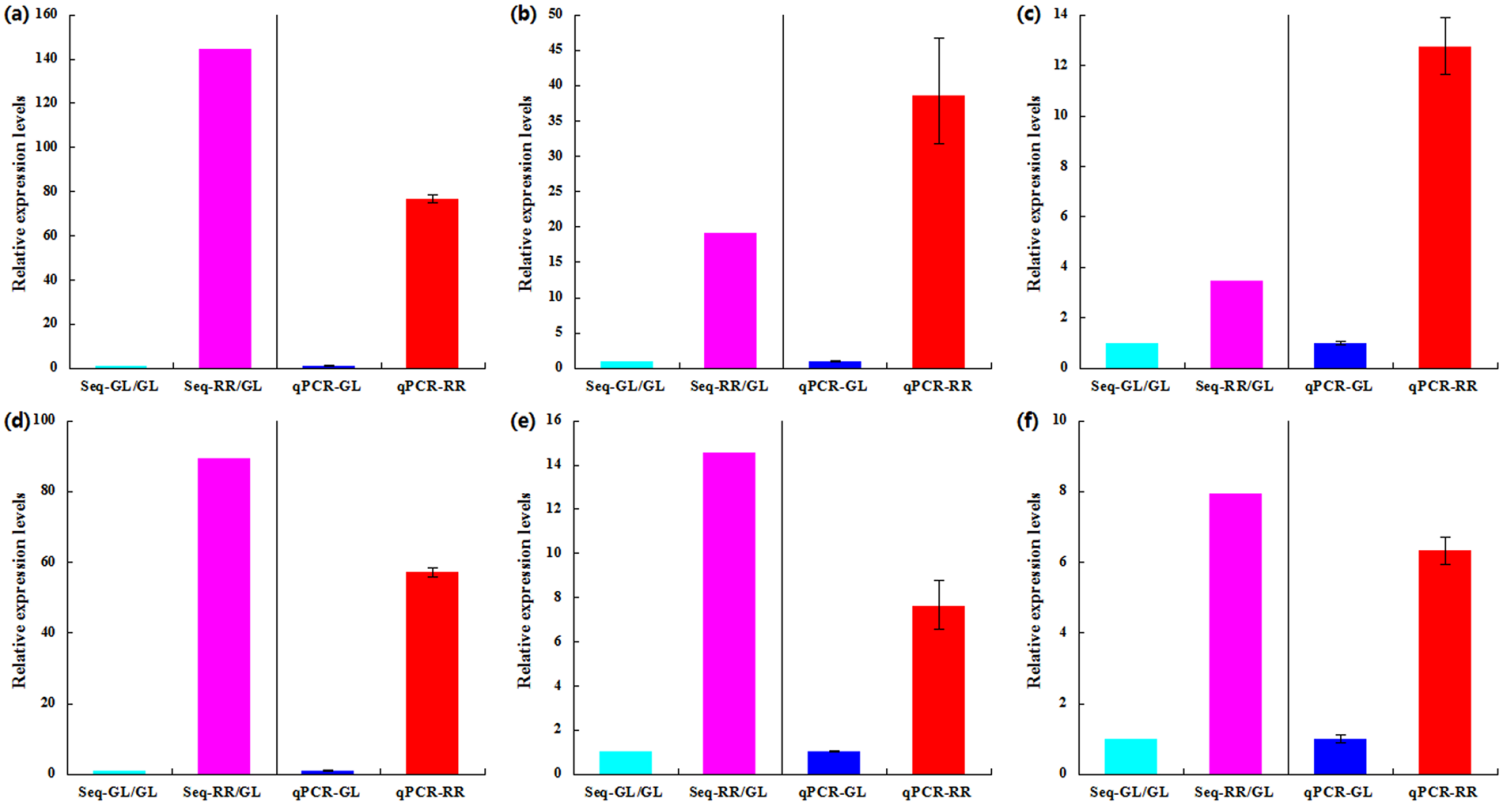
The qRT-PCR verification of the *G10H* and *12OPR* unigenes’ expression profiles between GL and RR lines in *Lithospermum erythrorhizon* (*Le*), *Arnebia euchroma* (*Ae*) and *Echium plantagineum* (*Ep*). (**a**) *G10H*-*LeCL2572*.*Contig1*, (**b**) *G10H*-*AeCL6292*.*Contig2*, (**c**) *G10H*-*EpCL2694*.*Contig1*, (**d**) *12OPR*-*LeCL2081*.*Contig2*, (**e**) *12OPR*-*AeCL94*.*Contig3*, (**f**) *12OPR*-*EpCL1162*.*Contig2*; Seq. data from RNA-seq, qPCR. data from qRT-PCR; The error bars in the qPCR results represent standard deviation of three biological replicates.

Furthermore, we verified the expression profiles of the *G10H* and *12OPR* unigenes in above three Lithospermeae plants by using qRT-PCR analysis. The qRT-PCR results were consistent with the results from RNA-seq data (Fig. 6). This indicated that *G10H* and *12OPR* unigenes should be up-expressed in all RR lines from above three Lithospermeae plants, and probably related to shikonin biosynthesis in this present study. Moreover, the functional validation of the *G10H* and *12OPR* unigenes is now underway, according to the similar strategy as reported in our previous papers^14, 31, 32^.

### Boraginales’ evolutionary status and its unigenes under positive selection (PSOs)

**Figure 7:**
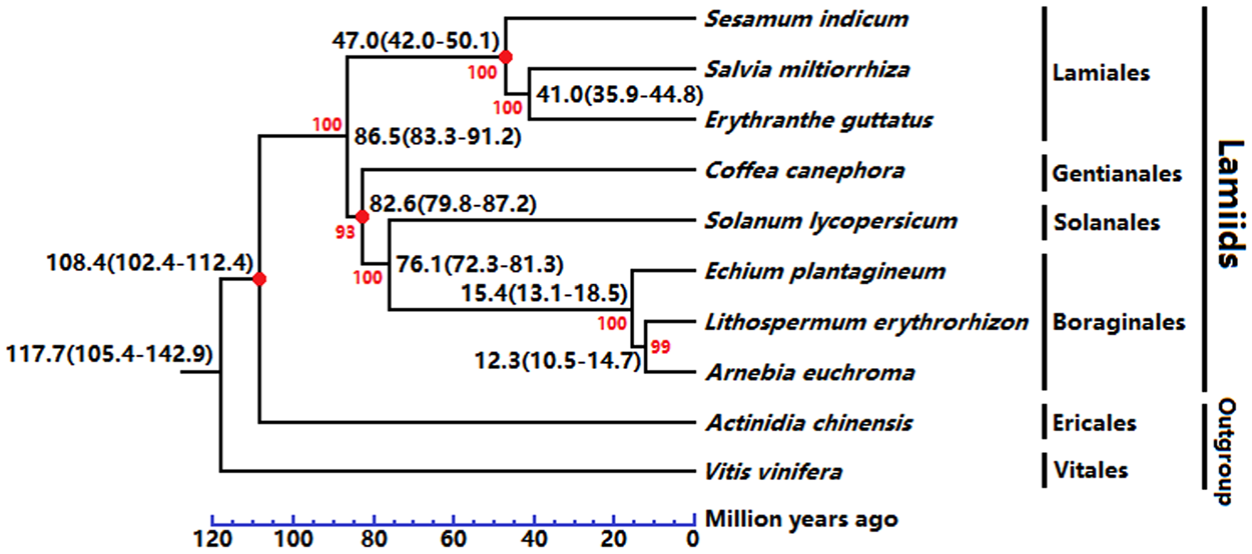
The phylogenetic model and divergence of Lamiids. (Black numbers) divergence times and their 95% confidence intervals, (Red numbers) maximum likelihood bootstrap support (MLBS), (Red nodes) divergence calibration points.

A total of 973 orthologous unigenes were identified from the above three Lithospermeae plants’ transcriptomes and seven plants’ known genomes (i.e., *Solanum lycopersicum*, *Coffea canephora*, *Salvia miltiorrhiza*, *Sesamum indicum*, *Erythranthe guttatus*, *Actinidia chinensis* and *Vitis vinifera*) (Supplementary Table S6). Based on these probable orthologous unigenes, a phylogenetic tree of Lamiids was established (Fig. 7). As shown in Fig. 7, the above three Lithospermeae plants (i.e., *L*. *erythrorhizon*, *A*. *euchroma* and *E*. *plantagineum*) and *S*. *lycopersicum* probably exhibit a more close evolutionary relationship than other species. This suggested Boraginales might be closer to Solanales/Gentianales than to Lamiales in the evolutionary distance scale. In addition, Boraginales probably segregated from Solanales at about 76.1 (81.3–72.3) million years ago (Mya), which is approximately at the Campanian (from 83.6 ± 0.7 Mya to 72.1 ± 0.6 Mya) in the late Cretaceous^33^.

According to previous studies, Boraginales’ evolutionary status in Lamiids is still unclear. For instance, Refulio-Rodriguez *et al*. analysed the phylogeny of Lamiidae based on nine plastid regions and one mitochondrial region in 129 samples; they inferred that Boraginales might be resolved as a sister to Lamiales, but that result had only 65% maximum likelihood bootstrap support (MLBS)^34^. As another example, Maximilian *et al*. presented a phylogenetic analysis of Boraginales with four chloroplast locations, including 90 samples; they deduced that Boraginales might be a sister group relationship to Solanales/Gentianales, but that result had only tentative statistical support^35^. Although there is a lack of genome/transcriptome of taxa samples in this present study, our result supported that Boraginales should be resolved as a sister to Solanales/Gentianales rather than Lamiales, because our results exhibited high MLBS (Fig. 7), and our results were based on sufficient orthologous unigenes (973, Supplementary Table S6) rather than a few of plastid regions/locations.

Furthermore, according to the above phylogenetic model of Lamiids, we identified that 145 orthologous unigenes were under positive selection (PSOs) in the above three Lithospermeae plants (Supplementary Table S6). Comparing their expression level in GL and RR lines separately, two orthologous unigenes (i.e. leucine-rich repeat kinase/extensin unigene (*LRE*) and 4-coumarate:CoA ligase unigene (*4CL*)) exhibited highly specific expression in all RR lines (Supplementary Table S6). *4CL* gene is directly involved in shikonin biosynthesis^8^. In the previous study, Yazaki *et al*. cloned two copies of *4CL* gene from *L*. *erythrorhizon* (i.e., *Le4CL*-*1* and *Le4CL*-*2*) and speculated that *4CL* gene might not significantly influence shikonin biosynthesis^8^. However, through PSO analysis combined with gene expression comparison, we deduced that some *4CL* unigenes (i.e., *LeCL15*, *AeCL1894* and *EpCL4063*) probably influence shikonin biosynthesis, although their expression level is relatively limited (Supplementary Table S4, Fig. 4).

## Materials and Methods

### Plants materials

Seeds of *L*. *erythrorhizon*, *A*. *euchroma* and *E*. *plantagineum* were germinated according to the methods, as previously reported^14, 26^. The germinated seeds were then transferred into square plastic pots (50 * 30 * 30 cm) with peat growing medium (Pindstrup, Denmark) and were cultured in a greenhouse at 23 ± 1 °C under a 16 h/day photoperiod for approximately 60 to 90 days. Subsequently, GL (green leaves/stems) and RR (red roots) samples from the above three Lithospermeae plants were collected, separately (Supplementary Table S7). All samples were immediately transferred into liquid nitrogen and stored in a ‒80 °C freezer. To minimize inter-individual differences, three biological replications of each sample were mixed together.

### HPLC analysis and the extraction of shikonin and its derivatives

Shikonin and its derivatives were extracted from all samples according to the following method. Each sample was ground in a grinding bowl with liquid nitrogen, and the ground powder was transferred into alcohol (99.7%, analytical reagent) at 25 ± 2 °C on a shaker at 120 rpm/min for 6 hours(samples:alcohol (w/v) = 0.1 g:2 mL)^26^. Subsequently, the sample was centrifuged at 10,000 g for 10 minutes. The supernatant was subject to HPLC analysis using an Agilent 1200 system (Agilent Technologies, USA). Separation was achieved using a Thermo Gold C_18_ column (4.6 × 250 mm, 5 μm, Thermo Fisher Scientific, USA). The mobile phase consisted of solvent A (HPLC water with 0.1% trifluoroacetic acid) and solvent B (acetonitrile) (A:B (v/v) = 30:70). The column oven temperature was at 40 °C; the flow rate was 1.0 mL/min; the injection volume was 10 μL; and the detection wavelength was at 515 nm, as previously reported^24–26^. Peaks were identified by comparing their retention times with the standard chromatogram of six shikonin and its derivatives (Supplementary Table S1), which were purchased from Nanjing PuYi Biological Technology CO., LTD (Nanjing, China).

### RNA extraction and sequencing

Total RNA was extracted using the TRIzol^®^ reagent (Invitrogen, Carlsbad, USA). The quality and quantity of the extracted total RNAs were detected and assessed using an Agilent 2100 Bioanalyzer (Agilent Technologies, USA) and a NanoDrop 2000 spectrophotometer (Thermo Fisher Scientific, USA) (Supplementary Table S8). Subsequently, cDNA libraries from each sample were prepared individually according to the following method: poly(A) enrichment, RNA fragmentation, cDNA synthesis, linker ligation, length selection, PCR purification and PCR amplification. Finally, the cDNA libraries were sequenced on an Illumina HiSeq 4000^TM^ platform, and the sequencing raw data were deposited in the NCBI’s Short Read Archive (SRA) under the following accession numbers: SRX1980116, SRX1980117, SRX2026182, SRX2026183, SRX2026190 and SRX2026191.

### Transcriptome assembly, annotation and CDS prediction

After raw data were filtered by removing adapter sequences, reads containing ploy-N, and low-quality sequences (Q < 20), the filtered clean reads were used to perform transcriptome *de novo* assembly using the Trinity program combining three independent software modules: Inchworm, Chrysalis, and Butterfly^36–38^. In addition, short contigs (<200 bp) were filtered automatically^36–38^. And then the CD-HIT-EST program was used for clustering assembled contigs to unigenes with an identity threshold of 95%^39^. Subsequently, the assembled unigenes were annotated through BLAST analysis against the seven public databases: NR (e-value of 1E^−5^); NT (e-value of 1E^−10^), SwissProt (e-value of 1E^−5^), InterPro (e-value of 1E^−5^), KEGG (e-value of 1E^−5^), COG (e-value of 1E^−5^) and GO (e-value of 1E^−5^)^40–44^. Furthermore, the unigenes’ translated amino acid sequences that could be mapped to functional annotation databases (in a priority order of NR, SwissProt, KEGG, COG) were defined as BLASTed coding sequences (CDSs); on the other hand, the unigenes that could not be aligned to any functional annotation databases were predicted as scanned CDSs by ESTScan software^45^.

### DEG identification

The expression level of each unigenes was calculated using RNA-Seq quantification analysis as the fragments per kb per million mapped fragments (FPKM) method^46, 47^. A rigorous algorithm was then used to identify differentially expressed unigenes (DEGs) based on the method of Audic and Claverie^48^. In addition, false discovery rate (FDR) was used to confirm the *p*-value in multiple tests^49, 50^. |Log_2_(ratio)| ≥ 1 and FDR < 0.001 were used as general thresholds to define significant differences in gene expression^48^, and |Log2(ratio)| ≥ 1 and FDR < 0.001 and Max(FPKM) ≥ 100 were used as stringent thresholds to define highly significant differences in gene expression in this present study.

### KEGG classification and enrichment

According to unigenes’ function in KEGG database, we classified all unigenes and DEGs, and then performed KEGG enrichment analysis for the pathways associated with each DEG using phyper modules from R software^40^. In addition, we calculate FDR for each *p*-value in KEGG enrichment analysis^49^. Finally, *p*-value < 0.05 was used as a threshold to define significant enrichment, becasue we found that the general threshold (FDR < 0.05) might be too stringent and not suitable in this present study.

### qRT-PCR verification

Total RNAs were extracted using TRIzol^®^ reagent (Invitrogen, Carlsbad, USA) and then treated with ReverTra Ace^®^ qPCR RT Kit (TOYOBO, Osaka, Japan) to reverse transcribe into cDNA. qRT-PCR was conducted by SYBR Green Realtime PCR Master Mix™ (TOYOBO, Osaka, Japan) on a BIO-RAD CFX Connect™ Optics Module system (BIO-RAD, USA). Three *G10H* copies, namely *LeCL2572*.*Contig1*, *AeCL6292*.*Contig2* and *EpCL2694*.*Contig1* (Supplementary Table S4), were selected for representing *G10H* unigenes in this study; and three *12OPR* copies, namely *LeCL2081*.*Contig2*, *AeCL94*.*Contig3* and *EpCL1162*.*Contig2* (Supplementary Table S5), were selected for representing *12OPR* unigenes in this study. The glyceraldehyde-3-phosphate dehydrogenase gene (*GAPDH*) was used as internal reference as previously reported^14, 32^. All primers were listed in Supplementary Table S8. Reactions were performed in three replicates. Three biological replicates were used to quantify relative gene expression levels, according to the 2^−ΔΔCt^ method^51^ (Supplementary Table S9).

### Orthologous genes identification, phylogenetic analysis and selection identification

Single-copy orthologous unigenes were identified by using InParanoid^52^ and MultiParanoid^53^ from the transcriptome data of above three Lithospermeae plants plus the public genome data of seven plants (i.e., *S*. *lycopersicum*, *C*. *canephora*, *S*. *miltiorrhiza*, *S*. *indicum*, *E*. *guttatus*, *A*. *chinensis* and *V*. *vinifera*; *A*. *chinensis* and *V*. *vinifera* are used as an outgroup)^54–60^. Subsequently, these orthologous unigenes were aligned and trimmed using PRANK and GBlocks programs^61, 62^. The tandem 4D-sites of the orthologous unigenes were then generated, and were used to support phylogenetic analysis and divergence time estimation. Then, the Phyml program was used to build a phylogenetic tree based on the maximum-likelihood (ML) method^63^. The divergence times were estimated and adjusted using MCMCtree and BEAST programs^64, 65^ based on the timing calibration points as previously reported^54–60^. A improved branch-site model that containing the null model (sites evolve under neutral or purifying selection) and the alternative model (sites evolve under positive selection on the foreground branch) was used to identify orthologous unigenes under positive selection^22, 66, 67^. The likelihood ratio test was used to distinguish the alternative model of each orthologous unigenes^22, 66, 67^. Finally, *p*-value < 0.05 was used as a threshold to define significant positive selection in this present study, becasue the general threshold (FDR < 0.05) might be too strict.

## Electronic supplementary material

Supplementary Dataset File

Supplementary Table S1

Supplementary Table S2

Supplementary Table S3

Supplementary Table S4

Supplementary Table S5

Supplementary Table S6

Supplementary Table S7

Supplementary Table S8

Supplementary Table S9
