# Supplementary material for: Transcriptome analysis explores genes related to shikonin biosynthesis in Lithospermeae plants and provides insights into Boraginales’ evolutionary history
Source: Sci Rep. 2017 Jun 30;7:4477. doi: 10.1038/s41598-017-04750-1 (PMC5493674; doi:10.1038/s41598-017-04750-1)
Supplement: Supplementary file 1 — Supplementary Dataset File [file 41598_2017_4750_MOESM1_ESM.doc]

**Transcriptome analysis explores genes related to shikonin biosynthesis in Lithospermeae plants and provides insights into Boraginales’ evolutionary history**

**Feng-Yao Wu^1,†^, Cheng-Yi Tang^1,†,b^, Yu-Min Guo^1^, Zhuo-Wu Bian^1^, Jiang-Yan Fu^1^, Gui-Hua Lu^1^, Jin-Liang Qi^1^, Yan-Jun Pang^1,c^, and Yong-Hua Yang^1,a^**

^1^State Key Laboratory of Pharmaceutical Biotechnology, NJU-NJFU Joint Institute of Plant Molecular Biology, School of Life Sciences, Nanjing University, Nanjing 210093, China.

^†^These authors contributed equally to this work.

^a^Email: yangyh@nju.edu.cn; ^b^Email: tangchengyi_nju@163.com; ^c^Email: pangyj@nju.edu.cn

**Supplementary Datasets**

**Supplementary Table S1.** Detection of shikonin and its derivatives in the RR lines by HPLC.

**Supplementary Table S2.** KEGG classification and enrichment analysis in *Lithospermum erythrorhizon*, *Arnebia euchroma* and *Echium plantagineum*.

**Supplementary Table S3.** Highly significant differentially expressed unigenes (HDEGs) in *Lithospermum erythrorhizon*, *Arnebia euchroma* and *Echium plantagineum*.

**Supplementary Table S4.** Unigene expression involved in shikonin biosynthesis in *Lithospermum erythrorhizon*, *Arnebia euchroma* and *Echium plantagineum*.

**Supplementary Table S5.** Unigene expression involved in methyl jasmonate biosynthesis in *Lithospermum erythrorhizon*, *Arnebia euchroma* and *Echium plantagineum*.

**Supplementary Table S6.** Orthologous unigenes and unigenes under positive selection (PSOs) from *Lithospermum erythrorhizon*, *Arnebia euchroma* and *Echium plantagineum*.

**Supplementary Table S7.** Plants and RNA samples.

**Supplementary Table S8.** The primers used in the qRT-PCR.

**Supplementary Table S9.** Quantification of relative expression levels and standard deviation (SD) in the qRT-PCR.
